# Association of Early-Life Family and Nieghborhood Circumstances With Depressive Symptoms in Chinese Older Adults

**DOI:** 10.1093/geroni/igab046.1707

**Published:** 2021-12-17

**Authors:** Chenkai Wu, Jie Tan

**Affiliations:** 1 Duke Kunshan University, kunshan, Jiangsu, China (People's Republic); 2 Duke Kunshan University, Kunshan, Jiangsu, China (People's Republic)

## Abstract

A growing body of literature suggests that early life circumstances can influence mental health throughout the lifespan. However, how these early life circumstances cumulatively contribute to depression in old age is not completely understood. The present study examined the associations of eight factors representing multifaceted early life experience at individual, family, and community levels with depression among community-dwelling older adults. Data were from the China Health and Retirement Longitudinal Study. We included 8,239 community-dwelling individuals who were ≥60 years, completed the life history questionnaire, and had assessment of depression. Chi-square test was used to examine the unadjusted associations between each of the eight early life risk factors and depression. An early life disadvantage index was established using risk factors that were significantly associated with depression. Logistic regression was used to examine the association of each early life risk factor and the index with depression. Of 8,239 individuals included, 2,055 (24.9%) had depression. In bivariate analysis, each of eight early life risk factors was significantly associated with depression. Except for maternal and paternal education, all risk factors persisted to be associated with depression after multivariable adjustment. In the multivariable-adjusted model, a one-point higher in the early life disadvantage index (range: 0-6) was associated with a 45% (95% CI: 37%, 53%) higher odds of depression. There was a strong association between early life environments and depressive symptoms among Chinese community-dwelling older adults. Adverse early life circumstances could contribute cumulatively to depression in old age.

